# Bon-Bons

**Published:** 1906-06

**Authors:** 


### Bon-Bons.

THE corporation of The Lyman D. Morse Advertising Agency, for many years at 38 Park Row, New York, and which is over sixty years old, is now The Morse International Agency, Revillon Building, 19 W. Thirtieth St., New York.

---

A HANDSOME BACTERIOLOGICAL WALL CHART, in colors, for physicians' office will be sent free to any of my readers upon request, mentioning the "Red Back." It is scientifically accurate and highly artistic and ornamental. Write to M. J. Breitenback Co., 53 Warren St., New York

"YES, colored physicians are eligible to membership in the American Medical Association, and there are colored physicians at present members of the American Medical Association."—*Dr. Geo. H. Simmons, Secretary, Editor Journal A. M. A.*

---

JUST THE THING I WANTED.—A nickle check-protector and envelope opener, sent me by Martin H. Smith & Co., 105 Chambers street, New York. A souvenir. They are not for sale, but are sent to "a few selected friends." Thanks.

---

NOT MUCH HURT.—The Golden Gate Advertising Co. of San Francisco has removed its offices temporarily to Reno, Nev. They write: "We were exceedingly fortunate, as not one of those connected with the business suffered any personal injury, and our pecuniary loss was only a nominal one." We extend congratulations.

---

"WHILE we are cutting out some of the publications that were heretofore in our advertising list, yours was one of the very first selected by the committee for the ensuing year."

F. E. TURNER,
"Registrar Medical Department, University, Nashville."
(See adv.—ED.)

---

DOCTORS, GO FISHING.—At the meeting of the San Angelo District Medical Society, May 29-30, ult., a novel feature of entertainment was introduced and much enjoyed. The whole party of physicians in attendance were driven to the river, where they camped and spent the night in fishing and in frying and eating black bass and the festive channel cat. Yum, yum!

---

A preliminary examination of applicants for appointment in the medical corps of the army will be held at various military posts throughout the United States on July 31, 1906. Full information in regard thereto may be obtained from the Surgeon General of the army, and applications must be filed prior to June 30. Thirty years is the prescribed maximum age, and persons whose age exceeds that limit are not eligible for examination.

M. W. IRELAND,
Major-Surgeon U. S. Army.

LETTER FROM SECRETARY RAMSAY.

ALTO, TEXAS, May 28, 1906.

*Dr. F. E. Daniel, Editor Texas Medical Journal, Austin, Texas.*

DEAR DOCTOR: The East Texas Medico-Chirurgical Association met in Palestine the 24th and 25th inst. with a good attendance and had a very fine meeting. There were several very able papers read and discussed thoroughly by quite a number. The Palestine physicians are a fine body of professional gentlemen and treat us so nice that we are always glad to go back; hence, our next meeting will be there in November next, and we hope it will be convenient for the editor of the good old "Red Back" to be on hand. We had hoped that you might find time to run up and spend a few hours with us this time. I am instructed to have the papers published in the TEXAS MEDICAL JOURNAL if you can find space for them during the year. Please write me in regard to it. The Association is in fine working order and promises to do great things for progressive medicine and surgery. Fraternally,

J. B. RAMSAY, Secretary.

[The papers will surely be published.—D.]

---

THE approach of the national holiday, July 4th, suggests to the surgeon the necessity of preparing to handle cases of cannon-cracker wounds and other injuries caused by the explosion of fire-works. Many of these, in the nature of things, will be infected with the bacillus of tetanus or its spores, and will require the most scientific treatment to save life. It is necessary only to review the files of this and other leading medical journals to gather a fair estimate of the enormous sacrifice of life that this country makes every year in celebration of Independence Day, and it behooves every medical practitioner to be prepared to receive and treat each case that presents itself with the best means at his command.

Without repeating the statistical facts that have been cited again and again in support of the prophylactic use of Antitetanic Serum, it is only necessary to say that these facts conclusively prove the value of the serum as a preventive of tetanus. It is injected in a single dose of 10 c.c. immediately after receipt of the injury, and should be repeated ten days later. The wound is to be thoroughly cleansed, avoiding the use of strong solutions or agents that coagulate the albumins, and packed with gauze well charged with Antitetanic Dusting Powder. Antitetanic Dusting Powder is Antitetanic Serum dried and powdered and mixed with a suitable quantity of Chloretone. It is recommended by reliable and experienced medical practitioners as a dressing for the wound in all cases in which tetanic infection is suspected. It is practically odorless and keeps well.

Antitetanic Serum and Antitetanic Dusting Powder are supplied by Messrs. Parke, Davis & Co., and may be obtained through all druggists.

---

THE MAN FOR LIEUTENANT GOVERNOR is Hon. F. F. Hill (Aubrey, Texas), Representative from Denton county in the last three Legislatures. I shall certainly vote for him, and I think every doctor in Texas should do the same. He invariably voted for the bills presented by the State Medical Association, not because they were asked for by the doctors, but for the stronger reason that the public welfare demanded their enactment. Dr. Blailock has withdrawn from the race, and it is now Hill and North Texas against Davidson, who makes in the public press a special bid for the doctors' support, and that, too, in face of his now notorious proposal to "let the doctors dissect each other."

